# Fluoroenesulphonamides: *N*-sulphonylurea isosteres showing nanomolar selective cancer-related transmembrane human carbonic anhydrase inhibition

**DOI:** 10.1080/14756366.2018.1461097

**Published:** 2018-04-30

**Authors:** Benoit Métayer, Andrea Angeli, Agnès Mingot, Kévin Jouvin, Gwilherm Evano, Claudiu T. Supuran, Sébastien Thibaudeau

**Affiliations:** a IC2MP-UMR CNRS 7582, Superacid Group–Organic Synthesis Team, Université de Poitiers, Poitiers, France;; b Department NEUROFARBA–Pharmaceutical and Nutraceutical Chemistry Section, Università degli Studi di Firenze, Sesto Fiorentino, Florence, Italy;; c Institut Lavoisier de Versailles, UMR CNRS 8180, Universitée de Versailles Saint-Quentin-en-Yvelines, Versailles, France;; d Laboratoire de Chimie Organique, Service de Chimie et Physico Chimie Organiques, Université Libre de Bruxelles, Brussels, Belgium

**Keywords:** Superacid, hydrofluorination, fluorinated isosters, carbonic anhydrase inhibitors, ureas

## Abstract

After hydrofluorination of ynesulphonamides in superacid or in the presence of hydrofluoric acid/base reagents, a series of α-fluoroenamides has been synthesised and tested for the inhibition of carbonic anhydrase (CA, EC 4.2.1.1) isoforms. This study reveals a new, highly selective family of cancer-related transmembrane human (h) CA IX/XII inhibitors. These original fluorinated ureido isosters do not inhibit the widespread cytosolic isoforms hCA I and II and selectively inhibit the transmembrane cancer-related hCA IX and XII, offering interesting new leads for future studies.

## Introduction

The elevated metabolic rate of solid cancer tumors leads frequently to acidosis and hypoxia^1^, which can be directly related to spatial disorganisation and flow-based disruption of an abnormal microvascularisation initiated by the growing tumor^2^. Under hypoxia stress exposition, tumor cells respond by transcription hypoxia inducible factor-1 (HIF-1α-activates), reprogramming their metabolism to overcome the reduced supply of oxygen^3^^,^^4^. The engaged nonoxygen-dependent glycolytic pathway results in increased production and export of lactic and carbonic acids to the extracellular proximal milieu, therefore decreasing extracellular pH^5^, which induces a variation of intracellular/extracellular pH ratio (pH_i_/pH_e_ ratio). This is regulated by different players including transmembrane carbonic anhydrases IX and XII (CA IX and CA XII) which are overexpressed in human cancer cells^6^. As a consequence, CA IX and CA XII are now recognised as especially relevant targets for cancer therapy. Sulphonamides and their bioisosteres (sulphamates, sulphamides, etc.) constitute the most investigated inhibitors of these enzymes^7^, with useful therapeutic applications^8^. They act on their deprotonated forms and bind the Zn^2+^ ion of the active site, disrupting the catalytic process^9^. However, this class of inhibitors suffers from side-effects that are directly related to the undesired inhibition of the cytosolic isoform I and II, abundant in many tissues and involved in numerous physiological functions^10^. As a consequence, numerous efforts were dedicated over the last years to the evaluation of non-zinc binding inhibitors. This resulted for example in the discovery that coumarins, thiocoumarines^11^ and, more recently, sulphocoumarins^12^, located at the entrance of the enzyme active site, were selective inhibitors of hCA IX isozyme. Our group recently contributed to this field by exploring the activity of tertiary benzenesulphonamides derivatives: substituted *N*-aryl-benzenesulphamides were found to act as selective nanomolar inhibitors of hCAs IX and XII^13^^,^^14^. Despite good affinity/selectivity to hCA IX and excellent stability in plasma, a study with their ^18 ^F-labelled analogues however showed no significant uptake in HT-29 tumors compared to normal organs/tissues^15^.

Considering the recent discovery of the urea derivative SLC-0111 which successfully ended Phase I clinical programmes for the treatment of patients with advanced hypoxic tumors over-expressing the isoforms hCA IX/XII^16^^,^^17^ and by the impact of ureas on pharmacokinetic properties, the evaluation of the corresponding sulphonylurea analogues must find interest. However, recent studies on the exploitation of bisarenesulphonylureas as anti-cancer agents led to unsatisfactory results in advanced clinical trials^18^^,^^19^, due to anemia and methemioglobinemia side effects that were correlated to the *in vivo* oxidative cleavage of the ureas and to the generation of the corresponding aniline-derived metabolites^20^^,^^21^. Nevertheless, sulphonylurea analogues of SLC 0111, where the sulphonyl ureido is considered as a linker, showed recently promising hCA IX and XII inhibitory properties^22^. In this study, coumarinyl-substituted analogues showed even more promising profile, with nanomolar inhibition of cancer-related hCA IX and XII and low micromolar inhibition of off-targets hCA I and hCA II. Exploiting a strategy commonly used in medicinal chemistry, the use of isosters of bioactive compounds^23^^,^^24^, we recently developed a method to design new fluoroenesulphonamides as *N*-sulphonylureas isosters^25^ and demonstrated their similarities^26^: these compounds, stable in solution, can be considered as good candidates to mimic unstable *N*-sulphonylureas. Therefore, following our seminal contribution on the use of tertiary benzene sulphonamides as selective cancer-related hCAs inhibitors, we considered that fluoroenesulphonamide group could represent an interesting novel selective chemotype and evaluated the activity of this new series against hCA I, hCA II, hCA IX and hCA XII.

## Materials and methods

### Chemistry

**Scheme 1.**
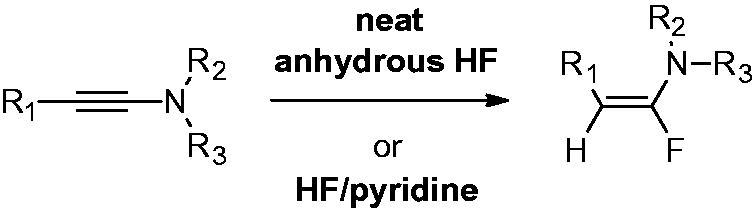
Hydrofluorination of ynamides.

Two methods were equally used to generate the fluroenamides from their corresponding ynamides, as shown in Scheme 1.

#### General procedure A

To a solution of HF (6 ml) maintained at −50 °C or −78 °C, was added very slowly ynamide derivative (1 mmol). The mixture was magnetically stirred at the same temperature during 5 min. The reaction mixture was then neutralised with water–ice–Na_2_CO_3_, extracted with ethyl acetate (×3). The combined organic phases were dried (MgSO_4_) and concentrated *in vacuo*. Products were isolated by column chromatography on silica gel.

#### General procedure B

To a mixture of hydrofluoric acid and pyridine (4 ml, 70/30 w/w) maintained at the required temperature was added the starting ynamide (1 mmol). The mixture was magnetically stirred at the same temperature during the required time. The reaction mixture was then neutralised with water–ice–sodium carbonate solution, extracted with dichloromethane (3×). The combined organic phases were dried over anhydrous magnesium sulphate, filtered and concentrated *in vacuo*. Products were isolated by column chromatography on silica gel. The NMR spectra of the products and their detailed characterisation can be found in literature^25^^,^^26^.

### CA inhibition assay

An SX.18Mv-R Applied Photophysics (Oxford, UK) stopped-flow instrument has been used to assay the catalytic activity of various CA isozymes for CO_2_ hydration reaction^27^. Phenol red (at a concentration of 0.2 mM) was used as indicator, working at the absorbance maximum of 557 nm, with 10 mM HEPES (pH 7.5) as buffer, and 0.1 M Na_2_SO_4_ (for maintaining constant ionic strength, which is not inhibitory against these CAs), following the CA-catalysed CO_2_ hydration reaction for a period of 10 s at 25 °C. The CO_2_ concentrations ranged from 1.7 to 17 mM for the determination of the kinetic parameters and activation constants. For each inhibitor, at least six traces of the initial 5–10% of the reaction have been used for determining the initial rate. The uncatalysed rates were determined in the same manner and subtracted from the total observed rates. Stock solutions of inhibitors (10 mM) were prepared in distilled deionized water and the solution diluted to 1 nM using the assay buffer. Inhibitor and enzyme solutions were pre-incubated together for 15 min (standard assay at room temperature) prior to assay, in order to allow for the formation of the enzyme–inhibitor complex. The inhibition constant (K_I_), was obtained by considering the classical Michaelis–Menten equation and the Cheng-Prusoff algorithm by using non-linear least squares fitting as reported earlier^28–30^.

## Results and discussion

A series of α-fluoroenesulphonamides and imides were therefore synthesised from the corresponding ynesulphonamides and imides according to our previously reported procedures^25–26^. *α*-Fluorenesulphonamide analogue **1** was therefore tested as a carbonic anhydrase inhibitor and found to be inactive toward hCAI and II, a poor micromolar inhibitor of HCAIX but, most interestingly, a nanomolar inhibitor of HCAXII. This result was especially encouraging as it reinforces our initial hypothesis and revealed a very selective inhibitor profile for the α-fluoroenesulphonamide pharmacophore (to be compared to acetazolamide reference compound AAZ, Table 1: entry 1).

**Table 1.** CA inhibition with acetazolamide (AAZ) as standard and compounds **1–7**, against isoforms hCA I, II, IX and XII, by a stopped flow CO_2_ hydrase adssay [27].

		Ki* (μm)*				Selectivity ratios			
Compound		hCA I^a^	hCA II^a^	hCA IX^b^	hCA XII^b^	I/IX	I/XII	II/IX	II/XII
	AAZ**	0.25^c^	0.012^c^	0.025^c^	0.006^c^	10.0	41.6	0.48	2
	**1**	/^e^	/^e^	>50	0.120	NC^f^	>1000	NC^f^	>1000
	**2**	/^e^	/^e^	>50	>50	NC^f^	NC^f^	NC^f^	NC^f^
	**3**	/^e^	/^e^	1.0	>50	>1000	NC^f^	>1000	NC^f^
	**4**	/^e^	/^e^	0.038	4.078	>1000	>1000	>1000	>1000
	**5**	/^e^	/^e^	0.021	0.366	>1000	>1000	>1000	>1000
	**6**	/^e^	/^e^	>10	0.116	NC^f^	>1000	NC^f^	>1000
	**7**	/^e^	/^e^	2.834	>50	>1000	NC^f^	>1000	NC^f^

* Errors in the range of ±5% of the reported data from three different assays.

** Acetazolamide (AAZ) was used as a standard inhibitor for all CAs investigated here.

a Recombinant isoforms, from Ref. [9a].

b Catalytic domain.

c Data collected from Ref. [9a].

d Not determined.

e Not active, K_I_ >100 µM.

f Not calculated.

To further explore substituent effect on the inhibitory activity/selectivity of this new class of hCA inhibitors, a brief structure activity relationships study was initiated. Exceptionally, all the tested fluoroenesulphonamides were found to be ineffective as offtarget hCA I and hCA II inhibitors and are selective inhibitor of the tumor associated isoforms IX and XII. Replacement of the phenyl ring on the alkene in **1** by a phenantrene or a thiophene (compounds **2** and **3**) revealed a strong influence of this substituent on the efficiency and selectivity of the inhibitors. Introduction of a phenanthrene (compound **2**) was indeed detrimental to the activity while the presence of a thiophene dramatically modified the inhibitory profile. 1-Thiophenyl-sustituted fluoroenesulphonamide **3** was found to be a micromolar inhibitor for hCA IX and not active for hCA XII and the presence of the heteroaromatic ring, in place of the tolyl group, shifts the inhibitor from a highly selective hCA XII inhibitor to a selective hCAIX inhibitor. These results suggest a non-zinc binding mode of action for these new chemotypes and evidence a variation of binding mode for these inhibitors^8^. To further verify this hypothesis, we next modified the position of the heteroatom in the thiophenyl substituent to impact eventual intra and inter molecular hydrogen bonding, analogously to what has been observed for aromatic ureas in solution^31^. In this case, compound **4** exhibited hCA IX nanomolar inhibition and low micromolar hCA XII inhibition. By increasing the distance between the fluoroenamide ureidoisoster moiety and the hydrophobic phenyl group, while maintaining a linear rigidity thanks to electronic conjugation between π electrons, a dual nanomolar selective inhibitor of hCAIX and hCAXII, compound **5**, could be discovered. Previous report of *in vivo* experiments nicely demonstrated that when silencing hCA IX alone leads to a 40% reduction in xenograft tumor volume, the concomitant inhibition of both transmembrane isoforms IX and XII leads to 85% reduction of tumor growth^6^. As a consequence, compound **5** can be considered as an interesting lead compound for further studies in this direction. To further explore the potential of fluoroenamide as a new chemotype for hCA selective inhibitors quest, fluoroenimides **6** and **7** were synthesised and tested. As for previously tested *N*-sulphonyl analogue **1**, compound **6** was shown to be nanomolar selective inhibitor of hCAXII. This result suggests that the α-fluoroolefin core, the ureido isoster, is the essential pharmacophore for these compounds, thus confirming our initial hypothesis. Again, shifting from phenyl to alkyl chains dramatically modify the selectivity of these compounds with compound **7** being now a hCA IX selective inhibitor at the micromolar level.

On the other hand, considering the substantial interest that bacterial/fungal/protozoan CA inhibition raised ultimately^32–34^, it would be of great interest to test some of these new CA inhibitors for their interaction with such enzymes belonging to other classes than the α-CAs investigated here.

## Conclusions

This study reveals a new, highly selective family of cancer-related transmembrane CA inhibitors. The tested α-fluoroenamides ureidoisosters did not inhibit widespread cytosolic isoforms hCA I and II, and selectively inhibited the transmembrane cancer-related ones, hCA IX and XII. The simple modification of the C-substituent of the α-fluoroenesulphonamide and α-fluoroenimide revealed the possibility to either generate selective hCA IX, selective hCA XII or dual hCA IX and hCA XII isoform confirming the strong potential of these new pharmacophores for further studies.
